# Occupation and metabolic syndrome: is there correlation? A cross sectional study in different work activity occupations of German firefighters and office workers

**DOI:** 10.1186/s13098-016-0174-0

**Published:** 2016-08-22

**Authors:** Markus Strauß, Peter Foshag, Bianca Przybylek, Marc Horlitz, Alejandro Lucia, Fabian Sanchis-Gomar, Roman Leischik

**Affiliations:** 1 Department of Cardiology, Sector preventive medicine, health promotion, Faculty of Health, School of Medicine, University Witten/Herdecke, Elberfelderstr. 1, 58095 Hagen, Germany; 2 Department of Cardiology, Faculty of Health, School of Medicine, University Witten/Herdecke, Urbacher Weg 19, 51149 Cologne, Germany; 3 Research Institute of the Hospital 12 de Octubre (“i+12”), Madrid, Spain; 4 European University of Madrid, Madrid, Spain

**Keywords:** Office worker, Firefighter, Metabolic syndrome, Metabolic risk factors, Metabolic risk

## Abstract

**Background:**

The treatment and prevention of the metabolic syndrome (MetS) is currently one of the major challenges in medicine. The impact of working conditions on metabolic risk has not been adequately studied. Our objective was to compare the prevalence of MetS and metabolic risk in two extremely different occupational groups: firefighters and office workers.

**Methods:**

A total of 143 male subjects (97 firefighters and 46 office workers) from Germany participated in the study. Anthropometric characteristics, metabolic risk parameters as well as laboratory parameters were collected. MetS was diagnosed according to criteria of the International Diabetes Federation.

**Results:**

Sedentary occupation showed a significant tendency towards obesity. Abdominal waist circumference was significantly greater in office workers than in firefighters [5.08 CI (1.44–8.71), p = 0.007]. Concerning metabolic risk factors, abnormal HDL, triglycerides, BMI, blood pressure and waist circumference values were more frequently found in office workers than in firefighters. The MetS was detected in almost 33 % of office workers as compared with only 14 % in firefighters (p = 0.015). Regarding MetS in an international comparison, the prevalence of MetS in German office workers was high and in firefighters it was extremely low.

**Conclusions:**

Sedentary occupation as an office worker is associated with a high risk of MetS. Both groups need to be made aware of the metabolic risks, and health promoting concepts such as corporate sports activities or education in healthy nutrition need to be implemented to counteract the development of the MetS and cardiovascular risk factors.

## Background

The prevalence of the metabolic syndrome (MetS) is steadily rising in the Western world and its prevention and treatment is a major challenge in medicine. It is estimated that in the year 2025, nearly 300 million people worldwide will be diagnosed with diabetes mellitus, a disease that is associated with MetS [1]. In Germany, the prevalence of the MetS is 23 %, with an upward trend [2]. Proportionally, the MetS is more common in men than in women [3].

The MetS comprises a constellation of interrelated metabolic disorders, including hypertension, central obesity, dyslipidemia atherosclerosis and hyperglycemia, that are associated with increased risk for cardiovascular disease, leading to increased morbidity and mortality [4–6]. Physical inactivity is also an important determinant for the MetS [7]. Although the working definition of the MetS varies among professional societies, all recognised definitions include a measure of obesity and insulin resistance.

The prevalence of the MetS and diabetes can be determinated by environmental setting (“healthy cities”), literacy and national health policy [8]. Approximately one and a half billion people worldwide are overweight and at least 400 million of them are obese [9]. In the PROCAM study, which examined the German population, 33 % of women and 35 % of men were overweight. Müller et al. [10] estimated an average body mass index (BMI) of the German population in 2040 of up to 30 kg/m^2^.

In this study, we compared metabolic risk in two working groups that differed widely in their activities: firefighters and office workers. In their daily work, firefighters are exposed to high physical and psychological stress [11–13], which is associated with high health risk [14, 15]. Therefore, good physical health is both necessary and expected as it reduces physical danger considerably. The second group comprised office workers in public administration, a numerically large workforce in Germany with approximately 18.5 million civil servants [16]. It is known that civil servants exhibit different health-risk behaviors and health forecasts according to the employment grade [17]. Working as an office worker in an administrative authority is characterized typically by sedentary work, which is associated with increased cardiovascular and metabolic risk factors [18, 19]. Indeed, studies from other countries have shown that working in an office environment is characterized by an unhealthy lifestyle and physical inactivity [20].

To date, little is known about the impact of working conditions for development of the MetS. It is therefore of major medical and socio-economic interest to examine different occupational groups regarding their metabolic risks and to compare the results in an international context.

## Methods

### Study population

We examined 143 male subjects (97 firefighters and 46 office workers). The firefighters group comprised full-time firefighters from fire departments of cities in the Westphalia area of Germany. Inclusion criteria were a full-time job as a firefighter and a regular participation in active emergency service. All firefighters worked on a shift schedule (shift duty 24 h/2–3 non-working days). The office worker group worked in the public administration of cities in the Westphalia area. The recruitment of study participants was performed on the basis of advertisements on workplace noticeboards and social media. Participation was voluntary.

### Information and informed consent

The study was approved by the Ethics Committee of the University of Witten/Herdecke (application no. 121/2013). Each subject was informed about participation in the study and gave their written consent.

### Examinations

Anthropometric parameters and medical history were collected by questionnaires. Body weight and body composition were determined using the Tanita BC-418MA Segmental Body Composition Analyzer (Tanita Corporation, Japan) [21]. A blood sample was obtained at the beginning of the examination to ascertain hematological parameters. Blood pressure measurements were performed using a calibrated standard blood pressure cuff with the subject in a supine position. Waist circumference was measured at the end of expiration while the subject was standing, using a measuring tape place around the waist at the midpoint between the lower edge of the ribs and the upper edge of the iliac crest. Diagnosis of the MetS was based on the criteria of the International Diabetes Federation (IDF) in 2005 [22].

### Statistical analysis

All statistical analysis was performed using Stata/IC 13.1 for Windows (StataCorp LP, College Station, TX). Categorical characteristics were described by specifying absolute and relative frequency. Anthropometric parameters, clinical and hematological characteristics parameters were described using mean, standard deviations (SD) and medians. Differences among groups were estimated using linear regression adjusted for age because most of the analyzed parameters were directly age-related; 95 % confidence intervals (CIs) are also reported. All statistical tests were two-sided with a significance level of 0.05.

## Results

### Age and professional experience

Table 1 shows the age and professional experience of the examined study population. Participating firefighters had an average age of 40.5 ± 9 years (range 23–58) and office workers had an average age of 45.8 ± 10 years (range 26–62). A significant difference was found between the age of the two groups [5.30 CI (1.86–8.74); p = 0.003]. Therefore, in subsequent comparisons the results were adjusted for age. No significant difference was found for the professional experience of the two groups. On average, professional experience of firefighters was 16.3 ± 9.1 years (range 2.5–37) and office workers 21.1 ± 10.8 years (range 3–46), taking into account any training period.

**Table 1:** Age and professional experience of the study population

	Firefighters				Office workers				Office worker vs. firefighter
	n	Mean	SD	Median	n	Mean	SD	Median	Estimated difference^a^ (95 %-CI) p value
Age (years)	97	40.5	9.0	41.0	46	45.8	10.0	48.0	5.30 (1.86 to 8.74) p = 0.003
Professional experience (years)	97	16.3	9.1	15.0	46	21.1	10.8	21.5	0.34 (−2.07 to 2.76) p = 0.779

^a^Linear regression adjusted for age

### Anthropometric characteristics

Table 2 describes anthropometric measurements of the two groups. No significant differences were found between the two groups in weight, height, BMI, muscle mass and body surface area. Although no significant differences were found between body fat, firefighters had a lower body fat percentage accompanied with a higher muscle mass than office workers. BMI in both groups was above the normal value of ≤25 kg/m^2^ and thus corresponded to pre-obesity.

**Table 2:** Anthropometric characteristics of the study population

	Firefighters				Office workers				Office worker vs. firefighter
	n	Mean	SD	Median	n	Mean	SD	Median	Estimated difference^a^ (95 % CI)/ p-value
Weight (kg)	97	85.9	11.5	85.1	46	87.1	13.4	83.8	0.13 (−4.38 to 4.64) p = 0.955
Height (cm)	97	182.2	6.3	182.0	46	181.5	5.4	182.0	−0.10 (−2.12 to 1.93) p = 0.924
BMI (kg/m^2^)	97	25.9	3.2	25.1	46	26.4	4.1	25.5	0.08 (−1.22 to 1.38) p = 0.904
Body surface area	97	2.08	0.16	2.09	46	2.09	0.17	2.05	0.00 (−0.06 to 0.06) p = 0.999
Muscle mass (kg)	97	67.1	6.9	67.2	46	65.3	6.4	65.7	−1.72 (−4.15 to 0.70) p = 0.162
Body fat (%)	97	17.7	6.2	17.5	46	20.8	6.5	20.3	2.05 (−0.23 to 4.33) p = 0.078

^a^Linear regression adjusted for age

### Metabolic risk factors

A significant difference was found in waist circumference, one of the central risk factors of metabolic syndrome, between firefighters and office workers [5.08 CI (1.44–8.71), p = 0.007]. Average abdominal waist circumferences was 89.8 cm in firefighters and 97.3 cm in office workers, which was above the threshold of ≥94 cm recommended by the IDF to define abdominal obesity. No significant differences were found for the remaining metabolic risk factors (Table 3).

**Table 3:** Metabolic risk factors for diagnosis of metabolic syndrome according to the IDF

	Firefighters				Office workers				Office worker vs. firefighter
	n	Mean	SD	Median	n	Mean	SD	Median	Estimated difference^a^ (95 % CI) p value
Central risk factors									
Waist circumference (cm)	97	89.8	10.0	90.0	46	97.3	11.7	94.5	5.08 (1.44 to 8.71) p = 0.007
BMI (kg/m^2^)	97	25.9	3.2	25.1	46	26.4	4.1	25.5	0.08 (−1.22 to 1.38) p = 0.904
Other risk factors									
Triglyceride (mg/dl)	97	142.2	75.1	126.0	46	162.1	91.4	130.0	9.55 (−19.37 to 38.47) p = 0.515
HDL (mg/dl)	97	55.5	12.7	53.0	46	55.8	14.8	54.5	0.61 (−4.18 to 5.40) p = 0.802
RRs_Rest_ (mmHg)	97	126.4	9.8	128.0	46	129.1	11.7	130.0	2.19 (−1.81 to 6.19) p = 0.281
RR_dRest_ (mmHg)	97	84.1	7.4	80.0	46	86.6	8.7	90.0	1.55 (−1.34 to 4.44) p = 0.292
HbA1c (%)	97	5.4	0.3	5.5	46	5.4	0.6	5.4	−0.09 (−0.25 to 0.07) p = 0.255

^a^Linear regression adjusted for age

Table 4 outlines the number and frequency of the presence of normal or abnormal values of risk factors of MetS according to IDF criteria. Statistically significant differences were found for the subdivision of the risk parameters in normal and abnormal waist circumference (p = 0.003) and HDL-value (p = 0.031), although these relationships were abolished after adjusting for age. The difference between the two groups by the factor waist circumference was clinically relevant (logistic regression adjusted for age p = 0.051).

**Table 4:** Number and frequency of the presence of normal or abnormal values of risk factors of metabolic syndrome according to the criteria of the IDF [22]

	Firefighters		Office workers		p value*	p value**
	Normal	Abnormal	Normal	Abnormal		
Triglyceride (mg/dl)	62 (63.9 %)	35 (36.1 %)	28 (60.9 %)	18 (39.1 %)	0.853	0.664
HDL (mg/dl)	94 (96.9 %)	3 (3.1 %)	40 (87.0 %)	6 (13.0 %)	0.031	0.070
RRs_Rest_ (mmHg)	49 (50.5 %)	48 (49.5 %)	17 (37.0 %)	29 (63.0 %)	0.152	0.195
RRd_Rest_ (mmHg)	58 (59.8 %)	39 (40.2 %)	21 (45.7 %)	25 (54.3 %)	0.150	0.296
Blood glucose (mg/dl)	94 (96.9 %)	3 (3.1 %)	42 (91.3 %)	4 (8.7 %)	0.212	0.613
Waist circumference (cm)	66 (68.0 %)	31 (32.0 %)	19 (41.3 %)	27 (58.7 %)	0.003	0.051
BMI (kg/m^2^)	87 (89.7 %)	10 (10.3 %)	40 (87.0 %)	6 (13.0 %)	0.777	0.831

* Exact Fisher-test

** Logistic regression adjusted for age

In all other risk factors (triglycerides, HDL, blood pressure, blood sugar, waist circumference, BMI) the values for firefighters were more often in percentage terms of the normal range than those of the office workers (Table 4).

### Metabolic syndrome

Before adjusting for age, the MetS was diagnosed significantly more often in office workers than in firefighters (p = 0.015). After adjusting for age, the MetS remained more common in office workers, although the difference was no longer significant (odds ratio 2.1 (0.8–5.2), p = 0.122). The MetS was diagnosed in nearly one out of three office workers (32.6 %) according to IDF criteria, but only in 14.4 % of firefighters (Table 5). The highest prevalence of MetS by firefighters was in the age group from 41 to 50 years (24.3 % of firefighters in this age group had a MetS), by office workers in the age group >50 years (38.9 %).

**Table 5:** Diagnosis of metabolic syndrome (number/frequency) according to the criteria of the IDF [22]

	n	No metabolic syndrome	Metabolic syndrome	p value (exact Fisher-test)
Firefighter	97	83 (85.6 %)	14 (14.4 %)	0.015
Office worker	46	31 (67.4 %)	15 (32.6 %)	
Prevalence of metabolic syndrome categorized in age groups				
Age	≤30	31–40	41–50	>50
Firefighter	0 (0 %)	2 (6.3 %)	9 (24.3 %)	3 (23.1 %)
Office worker	1 (16.6 %)	1 (20 %)	6 (35.3 %)	7 (38.9 %)

## Discussion

Abdominal circumference was significantly higher in office workers than in firefighters and therefore the prevalence of central obesity was more apparent. Furthermore, office workers had lower HDL cholesterol levels then firefighters, which was almost significant after adjusting for age. Other relevant factors, such as triglycerides, blood pressure and blood sugar levels did not significantly differ between office workers and firefighters. In the firefighter group, an increased waist circumference was found in 32 % of the subjects, whereas for office workers this increased to more than half (58.7 %). Central obesity can be detected by waist circumference or BMI by IDF defined criteria.

Regarding BMI, both groups were slightly above the normal range of 25 kg/m^2^, and no significant difference was found between the groups.

In this study, the definition of the International Diabetes Federation (IDF) was used to diagnose the MetS [22]; however, other definitions have been used by the World Health Organization (WHO) [23], the National Cholesterol Education Program (NCEP) [24], the American Heart Association (AHA) and the National Heart, Lung and Blood Institute (NHLBI) [25]. Overall, the prevalence of the MetS ultimately depends on the definition used. The use of the IDF definition of the MetS results in a higher prevalence of the MetS than other criteria [26]. The IDF definition for the MetS focuses on four criteria: obesity, dyslipidemia, hypertension and insulin resistance. For the diagnosis, the main criterion obesity and two more of the above criteria must be present.

Analyzing BMI for the diagnosis of overweight, only 10.3 % of firefighters in our study were overweight. When compared with the survey by Wilkinson et al. [27], where 82.5 % of all American firefighters were overweight or obese, the prevalence of overweight in German firefighters is markedly lower. High BMI values are also found in police officers, and may reflect a general problem in the public security sector [28]. However, several studies have found that waist circumference is a more precise measuring instrument for assessment of obesity than BMI [29]. Therefore, waist circumference measurement should be used for the diagnosis of obesity in firefighters [30, 31]. A similar result was found in a study by Lee et al. [32], who demonstrated that the MetS is independent of BMI. Nevertheless, BMI has a positive benefit for the assessment of general health status with firefighters because a high BMI is associated with a lower health status [33]. Similarly, the waist circumference for diagnosis of obesity in office workers seems to be more precise. By considering our examined office workers, a strong discrepancy between abnormal values of waist circumference and BMI was found (abnormal values waist circumference: 58.7 %, BMI: 13 %).

The significantly higher abdominal circumferences in office workers are likely due to sedentary work activities. Longer sedentary activities are associated with higher abdominal circumferences and the severity of a metabolic risk [34]. Long sedentary work is also linked with lower HDL cholesterol levels [18], and is consistent with our findings.

Using the IDF criteria, MetS was detected in 14.4 % of investigated firefighters and in 32.6 % of office workers. The prevalence of the MetS in firefighters in our study strongly correlates with the results of Donovan et al. [35], who reported that 15 % of American firefighters were diagnosed with MetS. However, two other studies from American firefighters showed a higher prevalence of the MetS [36, 37]. The question arises whether it is possible to transfer the findings of firefighters from other nations to the local conditions. It can be assumed that differences in professional activities and nutrition exist. It would be important to compare our study with other studies on German firefighters; however an extensive literature search yielded no results on other national studies, which points to neglect of the firefighting profession and this subject in Germany.

A literature review yielded no results on studies of the MetS in German office workers. In the general German population, a MetS prevalence of 32.7 % is described using the IDF definition [38]. The prevalence of MetS in the United States population is similar [39]. From 2003 to 2012, the overall prevalence of the MetS in the United States was 33 % (95 % CI 32.5 % 33.5 %) [39]. Our office workers represent a cohort of professionals with mainly sedentary work activity. Almost one third of all office workers presented an existing MetS. Other studies have already concluded that office working is characterized by an unhealthy lifestyle [19, 20]. Our office worker group displayed a higher health hazard, shown in a higher prevalence of MetS, similar to investigated office workers of Bangkok [40]. The effects of metabolic risk factors and their importance have been studied for many years, but it tends to be clinically under recognized [41]. In the future, the incidence of the MetS will continue to increase. To determine the impact of occupation on these factors, further studies (in Germany) are needed. Studies in other countries have already indicated that in some cases there are important differences in the prevalence of the MetS itself and between different occupational groups [39]. Table 6 shows a comparison of the prevalence of the MetS in our group with other professional groups in different countries.

**Table 6:** Prevalence of metabolic syndrome in published studies of firefighters and office workers

Prevalence of metabolic syndrome							
Firefighters				Office workers			
Study	Prevalence (%)	Country	Criteria	Study	Prevalence (%)	Country	Criteria
Own results	14.4	Germany	IDF	Own results	32.6	Germany	IDF
Title et al. [58]	19	Canada	Modified NCEP/ATP III	Lohsoonthorn et al. [40]	25.8	Thailand	Modified NCEP/ATP III
Carey et al. [37]	46.7	USA	National Institutes of Health Criteria 4	Konradi et al. [20]	34.6	Russia	IDF
Donovan et al. [35]	15	USA	ModifiedNCEP/ATP III	Suh et al. [59]	7.4	Korea	Modified NCEP/ATP III
Baur et al. [36]	28.3	USA	Modified criteria of joint scientific statements [25]	Matsuura et al. [60]	16	Japan	Japanese criteria [61]

A change in life-style practices is required to reduce metabolic risk. Impaired sleep rhythm is a factor which in turn is a risk factor for metabolic syndrome. Especially the eating behaviour is changed and interacts healthy nutrition and development of weight [42]. Night work causes a mismatch between the endogenous circadian timing system and the environmental synchronizers (light/dark cycle) [43, 44]. The Career Firefighters participated in this study work 24 h and have 3 non-working days. After that they work again 24 h a have again 3 non-working days. It might by presumed, that this shift work can change the nutrition habits and assisted to the development of metabolic syndrome [45–47]. It is well known that physical activity has a positive effect on metabolic risk factors [48–50]. Regarding the fire service, it has been proven that obese firefighters have a poorer health status and are more vulnerable to weight gain [51]. In addition, study results of American firefighters show significantly better cardiorespiratory fitness and less metabolic risk factors in normal weight versus overweight firefighters [52]. The enormous importance of cardiorespiratory fitness in relation to the metabolic system is demonstrated in a study of Baur et al. [36], who showed that the MetS was inversely proportional to cardiorespiratory fitness. These results underscore the importance of an approach for reducing the prevalence of the MetS in firefighters. It known that an increase in cardiorespiratory fitness is associated with a reduction in metabolic risk factors in firefighters [35]. Also, cardiorespiratory fitness is a necessary vital factor for admission to the firefighter service. Indeed, good cardiorespiratory fitness is a fundamental starting point for the promotion and maintenance of health in firefighters. Therefore, firefighters should try to improve their cardiorespiratory fitness through targeted training, thereby simultaneously minimizing metabolic risk factors. Examined office workers show a higher prevalence of MetS than those of the general German population. Office workers therefore seem to be particularly at risk for the development of MetS. It is absolutely necessary to reduce this risk with preventative measures. The first approach should be a change of lifestyle. This could be realized by increased physical activity and a change in diet. It has been shown that office workers do not compensate their sedentary lifestyles with physical activity during their leisure time [50, 53]. An investigation by Maruyama et al. [54] in 2010 showed a positive effect of physical activity with simultaneous participation in a nutrition program in “white collar workers” in relation to a reduction in body weight, BMI and fasting glucose levels.

## Conclusions

In conclusion, our results demonstrate that firefighters have lower metabolic risk than office workers. Shift work and stressful job conditions in professional firefighters might be more likely associated with increased cardiovascular risks [55, 56]. But, we cannot exclude the possibility that firefighters enjoy a healthier lifestyle. Firefighters presented less frequent abnormal values of all considered criteria for diagnosis of the MetS. Office workers had a significantly greater abdominal waist circumference than firefighters. Thus, it does not seem surprising that in our study the rate of the MetS was higher for office workers than firefighters. Previous research has also described a difference of metabolic risk between different occupational groups and there is mounting evidence that a link between the metabolic risk and the occupation exists. Occupational groups need to be made aware of the risk and health promoting concepts have to be implemented in daily lives, such as corporate sports activities or nutrition education. Further studies are required to determine the occupational impact on health different local conditions.

## Limitation

The ratio of waist to hip circumference ratio would provide a further information about the metabolic syndrome and cardiovascular risks in firefighters and office workers [57]. In the present study we did not measure this factor. For future scientific work this ration has to be considered.

## Abbreviations

BMI: body mass index
HDL: high-density-lipoprotein
IDF: International Diabetes Federation
MetS: metabolic syndrome
